# Mugwort Leaf Powder (*Artemisia argyi*) Alleviates Lipid Metabolism Disorders and Intestinal Health of Common Carp (*Cyprinus carpio*) Fed High-Fat Diets

**DOI:** 10.1155/anu/3972704

**Published:** 2025-11-03

**Authors:** Xinxin Xu, Yue Xi, Yuru Zhang, Xianglin Cao, Ronghua Lu, Guoxing Nie

**Affiliations:** College of Fisheries, Henan Normal University, Xinxiang, Henan 453000, China

**Keywords:** common carp, intestinal health, lipid metabolism, mugwort leaf powder

## Abstract

Mugwort leaf powder (MLP), as a typical Chinese herbal medicine containing lots of flavonoids and polysaccharides, has strong anti-inflammatory and immune effects. However, there are relatively few studies on the use of MLP in aquatic animals fed high-fat (HF) diets (HFDs), especially in terms of lipid metabolism and intestinal health. To investigate the impacts of MLP on the growth performance and health status of common carp fed HFDs, common carp were provided with a basal diet, an HFD, and the HFDs enriched with 0.6%, 1.2%, 1.8%, and 2.4% MLP for 8 weeks. Dietary supplementation with 0.6%−1.2% MLP enhanced fish growth and feed utilization compared to the HF group (*p* < 0.05). Supplementation with over 1.2% MLP increased villus height and digestive enzyme activities (*p* < 0.05). Supplementation with 1.2% MLP upregulated intestinal anti-inflammatory gene expression and decreased serum triglyceride levels (*p* < 0.05). Moreover, MLP significantly mitigated the degree of hepatocyte vacuolation and reduced adipocyte size (*p* < 0.05). Supplementation with over 1.2% MLP decreased the crude lipid, triglyceride, and cholesterol levels in the hepatopancreas, as well as downregulated the lipid synthesis gene expression of *fas* and upregulated the lipolysis gene expression of *cpt1* in the hepatopancreas (*p* < 0.05). The gene expression related to lipid synthesis in adipose tissue also exhibited a downregulated tendency (*p* < 0.05). Combining the quadratic regression results of the above indices, this study concluded that supplementation with 0.92%−2.17% MLP positively influenced the growth, intestinal well-being, and lipid metabolism of common carp fed HFDs.

## 1. Introduction

Lipids serve as vital nutrients for aquatic species, significantly contributing to their nutrient metabolism [1]. Research indicates that aquatic animals can efficiently utilize lipids, with their capacity for lipid utilization significantly surpassing that for proteins and carbohydrates. Thus, lipids serve as a more effective energy source for aquatic animals [2], and high-fat (HF) diets (HFDs) can exhibit a significant protein-sparing effect, enhancing feed efficiency, reducing feed costs, and lowering nitrogen emissions [3]. Consequently, the widespread adoption of HFDs is a crucial strategy in the intensive farming practices of the aquaculture industry [4]. However, prolonged feeding of HFDs can result in excessive lipid accumulation in the liver of cultured fish, leading to liver damage and lipid metabolism disorders. This can severely impair the growth, reduce the immunity, and lead to disease outbreaks, ultimately causing substantial economic losses in cultured fish [5, 6]. Therefore, it is essential to find functional substances that can mitigate the adverse effects of HFDs on fish health. Medicinal plants have been a vital resource for human health for thousands of years [7]. A report from the World Health Organization (WHO) suggests that, as the quality of life for individuals improves, the global demand for medicinal plants has been increasing. Around 80% of the global population relies primarily on unconventional medicines, particularly Chinese herbs, for the initial treatment of diseases [8, 9]. Similarly, the use of Chinese herbs is also prevalent in the prevention of animal diseases and the application as feed additives [10].

Mugwort leaf (*Artemisia argyi*), a perennial herb of the Asteraceae family, is widely distributed across various regions, including Asia, America, and Europe [11–13]. Mugwort leaf can be utilized as a food source due to their distinctive taste and aroma. For instance, it is often prepared as green balls with glutinous rice and is commonly featured in various special festivals [14–16]. Moreover, mugwort is also treated as a traditional edible herbal medicine and boasts a long history of medicinal use [17]. The bioactive compounds present in mugwort, such as flavonoids, volatile oils, polysaccharides, phenylpropanoids, and triterpenoids, contribute to its significant antibacterial, antiviral, and anti-inflammatory properties [18]. Research has demonstrated that mugwort leaf extract can enhance growth, boost antioxidant activity, enhance immune function, and affect serum lipid metabolism in mice [19–21]. Mugwort leaf powder (MLP) is also widely used in the poultry and livestock breeding industry to stimulate appetite, enhance coloration, improve the quality of animal products, promote growth, optimize production performance, and reduce disease incidence [22]. However, there are relatively few studies examining the effects of mugwort leaf on aquatic animals as a whole. For instance, a mixture of mugwort and *Houttuynia cordata* Thunb has been shown to enhance antioxidant activity, improve hepatic lipid metabolism, and alter gut bacteria composition in grass carp (*Ctenopharyngodon idella*) [23]. Mugwort leaf essential oil has been shown to repair liver damage in zebrafish (*Danio rerio*) by regulating the composition of intestinal flora [24]. The water extract of mugwort leaf has been shown to improve growth performance and hepatopancreas health in common carp (*Cyprinus carpio*) [25]. These studies suggest that mugwort leaf may have a regulatory effect on the lipid metabolism of aquatic animals.

The common carp, acknowledged as a crucial species in freshwater aquaculture across Asia, is widely appreciated by consumers for its delicious meat and rich nutritional profile [26]. However, prolonged feeding with HFDs may negatively impact lipid deposition, a concern prevalent in the intensive farming of common carp [6]. Considering the potential benefits of mugwort leaf in animal nutrition, this research intends to explore how the inclusion of MLP in HFDs influences fish growth, lipid synthesis and decomposition, and intestinal health.

## 2. Materials and Methods

### 2.1. Ethics Statement

The experiment was conducted in compliance with established animal welfare and ethical standards of China and received approval from the Animal Care and Use Committee at Henan Normal University (Permission Number: HNSD-2024BS-1101).

### 2.2. Diets Designing and Fish Management

A control diet (CT, 5% lipid diet) and five HFDs (10% lipid diet) supplemented with 0%, 0.6%, 1.2%, 1.8%, and 2.4% MLP, referred to as HF, MLP1, MLP2, MLP3, and MLP4 groups, respectively, were prepared at the aquaculture base of Henan Normal University (Henan, Xinxiang), following the method described by Xu et al. [6]. In brief, the raw materials, including soybean meal, cottonseed meal, and rapeseed meal, were initially crushed. These materials were mixed step by step with other powdered ingredients. A specific proportion of water and soybean oil, as outlined in the formula, was then added, allowing for granulation after thorough mixing. The prepared diets were air-dried and stored at −20°C. Among these ingredients, the MLP was purchased from Tangyin North Aian Tang North Ai Company (Henan, Anyang), and the carbohydrate, protein, and fat raw materials were provided by Henan Normal University (Henan, Xinxiang).

Experimental fish were sourced from the aquaculture base of Henan Normal University and temporarily raised for 2 weeks in the recirculating aquaculture system at the same facility. A total of 540 healthy juvenile fish were randomly (6 groups, 3 replicates/group, 30 fish/replicate) fed 6 different experimental diets over a period of 8 weeks. Feeding occurred three times daily (8:00, 12:00, 16:00). The water quality parameters during the feeding period were as follows: the concentration of dissolved oxygen was maintained above 8 mg/L, with the pH ranging from 7.3 to 7.7. Water temperature was sustained at 28 ± 2°C, and the light was 12 h of light/12 h of darkness. Additionally, the mortality rate of the experimental fish was monitored and recorded during the feeding period.

### 2.3. Sampling

Prior to sampling, a 24 h fasting period was imposed on the fish. Subsequently, the experimental fish were anesthetized using MS-222. The fish quantity in each barrel was recorded, along with their weight and body length. Nine fish from each barrel were chosen randomly for blood sampling. The chosen blood samples were kept at 4°C for 6 h before being centrifuged at 3500 rpm for 10 min. Serum was collected in a sterile, enzyme-free EP tube and preserved at −80°C. The viscera, including the hepatopancreas, spleen, kidney, adipose tissue, and intestine, were separated and weighed, while the length of the intestine was recorded. Samples of hepatopancreas and muscle were collected and preserved. Additionally, tissues were rapidly isolated from nine fish randomly selected from each tank and stored at −80°C. Hepatopancreas, adipose tissue, and intestinal tissues from three fish were randomly selected from each barrel and preserved in a 4% paraformaldehyde solution. Finally, nine fish from each barrel were directly stored at −20°C.

### 2.4. Sample Detection

#### 2.4.1. Nutritional Composition and Fatty Acid (FA) Composition of Samples

The nutritional composition of diets, whole fish, hepatopancreas, and muscle was measured according to the method description of the Association of Official Analytical Chemists [27]. The extraction of FAs of diets and different tissues was briefly described as follows: the lipid portion of the sample extracted using a chloroform-methanol solution was dissolved in n-hexane, followed by thorough mixing with a 0.4 mol/L potassium hydroxide-methanol solution. Subsequently, distilled water was added, and the mixture was allowed to stratify. The mixture was then centrifuged at 3000 rpm for 5 min. The supernatant was collected and then analyzed by gas chromatography by an Agilent 7820A Series GC device (Agilent Technologies, Santa Clara, CA, USA) [28].

**Figure 1:**
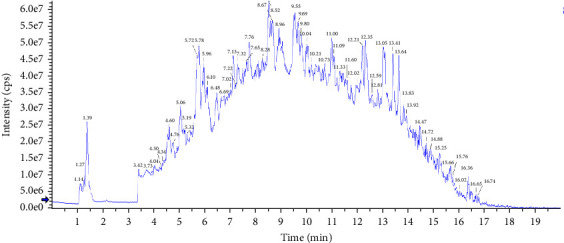
Positive ion chromatograms of MLP obtained by UHPLC-MS/MS.

The nutritional composition and FA composition of diets are shown in Tables 1 and 2, respectively. The nutritional composition of MLP was determined using UHPLC-MS/MS by Hefei Kexiaoyan Technology Co., Ltd (Anhui, Hefei), the results are shown in Table 3 and Figure 1.

#### 2.4.2. Biochemical Parameters

The measured serum parameters encompassed alanine aminotransferase (ALT, Assay Kit Number: C009-1-1), aspartate transaminase (AST, Assay Kit Number: C010-1-1), total cholesterol (TC, Assay Kit Number: A111-2-1), triglycerides (TGs, Assay Kit Number: A110-2-1), glucose (GLU, Assay Kit Number: F006-1-1), high-density lipoprotein (HDL, Assay Kit Number: A112-2-1), low-density lipoprotein (LDL, Assay Kit Number: A113-2-1), total antioxidant capacity (T-AOC, Assay Kit Number: A015-1-2), superoxide dismutase (SOD, Assay Kit Number: A001-1-2), catalase (CAT, Assay Kit Number: A007-1-1), and malondialdehyde (MDA, Assay Kit Number: A003-1-2) levels. Additionally, the hepatopancreas was assayed for TG, TC, and nonesterified FA (NEFA, Assay Kit Number: A042-1-1) levels. Furthermore, intestinal tissue was analyzed for trypsin (Assay Kit Number: A080-2-2), lipase (Assay Kit Number: A054-1-1), and amylase activities (Assay Kit Number: C016-1-1). All measurements were performed according to the guidelines specified in the kits of Nanjing Jiancheng Bioengineering Institute (Jiangsu, Nanjing).

#### 2.4.3. Histological Observation of Tissues

After the fixed tissue samples, including hepatopancreas, adipose tissue, and intestinal tissues, were dehydrated through a graded ethanol series and then embedded in paraffin, serial sections were prepared using a paraffin microtome. The sections were then dried, stained with hematoxylin and eosin (H&E), sealed, and subsequently observed and photographed under an optical microscope. The morphological observation and statistical analysis were performed based on the methods described by Xu et al. [29] with minor modifications. The morphological parameters of the intestinal tissue were quantified as follows: the height and width of all intestinal villi in each section, and the muscle thickness at four different locations in randomly selected sections were statistically analyzed.

#### 2.4.4. Real-Time Quantitative PCR (RT-qPCR) Measurement

RNA was extracted from the hepatopancreas, adipose tissue, and intestinal tissues by TRIZOL (Vazyme, Nanjing). After determining the concentration and purity of the total RNA, complementary DNA (cDNA) was synthesized using the PrimeScript RT kit (Vazyme, Nanjing). RT-qPCR was performed using a CFX-384 RT-qPCR system. The relative mRNA expression was calculated using the 2^−ΔΔCT^ method [30]. The sequences of reference genes and primers are listed in Table S1.

#### 2.4.5. Statistical Analysis

All data collected in the experiment were analyzed using SPSS 26.0 software. The results were expressed as mean ± SEM. The significance of differences was determined using one-way analysis of variance (ANOVA) followed by the Duncan multiple comparison test, with a significance level set at *p* < 0.05. The primary and secondary regression models were developed with dietary MLP supplement levels (0%, 0.6%, 1.2%, 1.8%, and 2.4%) as the independent variable in HFDs. *p*-Values for the models were calculated by SPSS 26.0, while GraphPad Prism 8 was used for plotting. The formulas for growth performance, feed utilization, and morphological indexes are shown in Table S2.

## 3. Results

### 3.1. The Main Composition of MLP

To determine the chemical composition of MLP, UHPLC-MS/MS analysis was performed. The ion chromatogram fingerprints of MLP acquired in positive ion mode are presented in Figure 1. This study successfully identified 42 main bioactive components in MLP using UHPLC-MS/MS technology, including flavonoids (luteolin, apigenin), flavonoid glycosides (rutin, diosmin), phenolic acids (caffeic acid), and coumarins (umbelliferone). The results demonstrated that MLP exhibited significant antioxidant and anti-inflammatory activity. The detailed chromatographic characterization is shown in Table 3.

### 3.2. MLP Improved the Growth Performance of Fish Fed With HFDs

The fish in the HF group showed significantly lower FBW, WGR, and SGR compared to those in the CT group (*p* < 0.05). In contrast, the FBW and WGR of fish belonging to MLP1 and MLP2 groups exhibited an upward trend, whereas the SGR in the MLP1 group was significantly higher than in the HF group (*p* < 0.05). The FCR in the MLP1 group was significantly lower than that of the HF group (*p* < 0.05). Furthermore, SI in the MLP4 group was significantly higher than in the HF group (*p* < 0.05). No significant differences were noted in the other indices (*p* > 0.05) (Table 4).

### 3.3. MLP Optimized the Body Composition of Fish Fed With HFDs

The moisture of whole fish in the HF group was considerably greater compared to the CT group. In contrast, the moisture contents in the MLP1, MLP2, and MLP3 groups were significantly lower than those in the HF group (*p* < 0.05). Additionally, the crude lipid content of whole fish in the HF group was significantly higher than that found in the CT group, with the inclusion of MLP further decreasing the crude lipid contents in MLP groups (*p* < 0.05). Conversely, the crude lipid content in the muscle of MLP groups did not differ significantly from that of the HF group (*p* > 0.05). Additionally, no significant differences were observed in the crude protein and ash content of whole fish, nor in the moisture, protein content, and ash content of muscle tissue among all groups (*p* > 0.05) (Table 5).

### 3.4. MLP Affected the FAs Composition of Tissues

The contents of SFA and MUFA in the whole fish, muscle, and hepatopancreas of the HF group were significantly reduced, while n-6 PUFA contents were increased compared to the CT group (*p* < 0.05). The n-3 PUFA contents in the whole fish and muscle of the HF group were significantly higher than those in the CT group (*p* < 0.05). The MLP addition groups demonstrated significantly higher contents of C18:3n-3 in the whole fish, muscle, and hepatopancreas compared to the CT group, with the highest content observed in the hepatopancreas of the MLP3 group (*p* < 0.05). In the hepatopancreas, n-3 PUFA contents in the HF, MLP2, MLP3, and MLP4 groups were lower than those in the CT group (*p* < 0.05), while no significant difference was observed between the MLP1 and the CT group. A significant decrease in DHA content was noted in the hepatopancreas of the HF group when compared to the CT group; however, this trend was reversed in the MLP1 group (*p* < 0.05). The n-6 PUFA contents in the hepatopancreas of the MLP1 and MLP2 groups were significantly lower than those in the HF group (*p* < 0.05). However, the n-6 PUFA and DHA contents in the whole fish and muscle of the MLP supplementation groups did not differ significantly from those in the HF group (*p* > 0.05) (Tables 6, 7, and 8).

### 3.5. MLP Enhanced the Serum Lipid Regulatory Capacity and Antioxidant Capacity of Fish Fed With HFDs

The levels of ALT and AST in the serum of the HF group were significantly higher than those in the CT group (*p* < 0.05). After the addition of MLP, the serum levels of ALT and AST in the MLP groups were significantly lower than those in the HF group (*p* < 0.05). Additionally, the serum levels of TC and LDL in the HF group were increased compared to those in the CT group (*p* < 0.05). Following the incorporation of MLP, the TC level in the MLP3 group was significantly lower than that in the HF group, and the LDL levels in the MLP groups were also significantly lower than those in the HF group (*p* < 0.05). Furthermore, the TG levels in the MLP groups exhibited a downward trend, with the MLP2 group showing a significantly reduced TG level compared to the HF group (*p* < 0.05). No significant differences were observed in the levels of GLU and HDL among all groups (*p* > 0.05) (Table 9).

The serum T-AOC level in the HF group was lower than that in the CT group (*p* < 0.05). The T-AOC levels in the MLP2 and MLP3 groups were higher than those in the HF group (*p* < 0.05). Additionally, the serum MDA level in the HF group was significantly increased compared to those in the CT group, while MDA levels in the MLP2 and MLP3 groups were reduced compared to the HF group (*p* < 0.05). There were no significant differences in SOD and CAT levels among all groups (*p* > 0.05) (Table 9).

### 3.6. MLP Strengthened Intestinal Health of Fish Fed With HFDs

**Figure 2:**
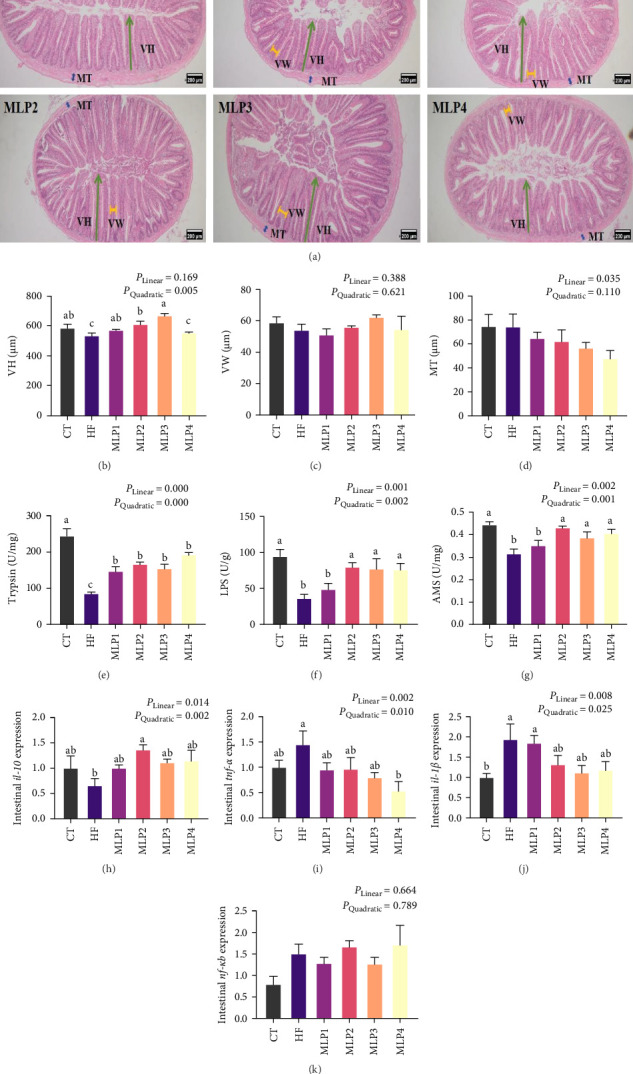
Effects of MLP supplementation in HFDs on intestinal health of common carp (*n* = 3); (A) The observation of intestinal tract (10x). Yellow line means villus width (VW), green line means villus height (VH), blue line means muscle thickness (MT). (B–D) The statistics results of intestinal observation: (B) villus height, (C) villus width, and (D) muscle thickness. (E–G) Intestinal digestive enzyme activities of common carp: (E) trypsin activity, (F) lipase (LPS) activity, and (G) amylase (AMS) activity. (H–K) The gene expressions of inflammation in the intestinal tissue of common carp: (H) *il-10* (*interleukin-10*) relative mRNA expression, (I) *tnf-α* (*tumor necrosis factor-α*) relative mRNA expression, (J) *il-1β* (*interleukin-1*β) relative mRNA expression, and (K) *nf-κb* (*nuclear Factor kappa-B*) relative mRNA expression. CT, control group; HF, high lipid group; MLP1, the HFD supplemented with 0.6% MLP group; MLP2, the HFD supplemented with 1.2% MLP group; MLP3, the HFD supplemented with 1.8% MLP group; MLP4, the HFD supplemented with 2.4% MLP group. Values are means with SEM represented by vertical bars. Different superscript letters (a, b, c) indicate statistically significant differences among groups (*p* < 0.05). *p*-Values for the linear and quadratic relationships with dietary MLP levels were determined by orthogonal polynomial analysis.

From the observation of intestinal morphology, it was found that the intestinal villi were intact and regularly arranged among all groups (Figure 2A). The intestinal villi height in the HF group was significantly lower than that in the CT group (*p* < 0.05). The addition of MLP significantly increased the intestinal villi height in the MLP1, MLP2, and MLP3 groups (*p* < 0.05). However, when the concentration of MLP reached 2.4%, a decrease in intestinal villi height was observed (*p* < 0.05). No significant differences were found in intestinal villus width or muscle thickness among the groups (*p* > 0.05) (Figure 2B–D).

The activities of trypsin, lipase, and amylase in the HF group were decreased (*p* < 0.05). When the supplemental level of MLP exceeded 1.2%, the activities of intestinal digestive enzymes in the MLP2, MLP3, and MLP4 groups were significantly higher than those in the HF group (*p* < 0.05) (Figure 2).

The expression of proinflammatory factors *il-1β* and *tnf-α* in the HF group was significantly upregulated compared to the CT group (*p* < 0.05). Moreover, the gene expression of both *il-1β* and *tnf-α* exhibited a downward trend with the MLP supplement, with the *tnf-α* expression in the MLP4 group being significantly lower than that in the HF group (*p* < 0.05). The gene expression of the anti-inflammatory factor *il-10* was upregulated in the MLP groups, particularly in the MLP2 group, which showed a significantly higher expression compared to the HF group (*p* < 0.05). No significant differences were found in the expression of the proinflammatory factor *nf-κb* across all groups (*p* > 0.05) (Figure 2).

### 3.7. MLP Improved Lipid Metabolism of Fish Fed With HFDs

**Figure 3:**
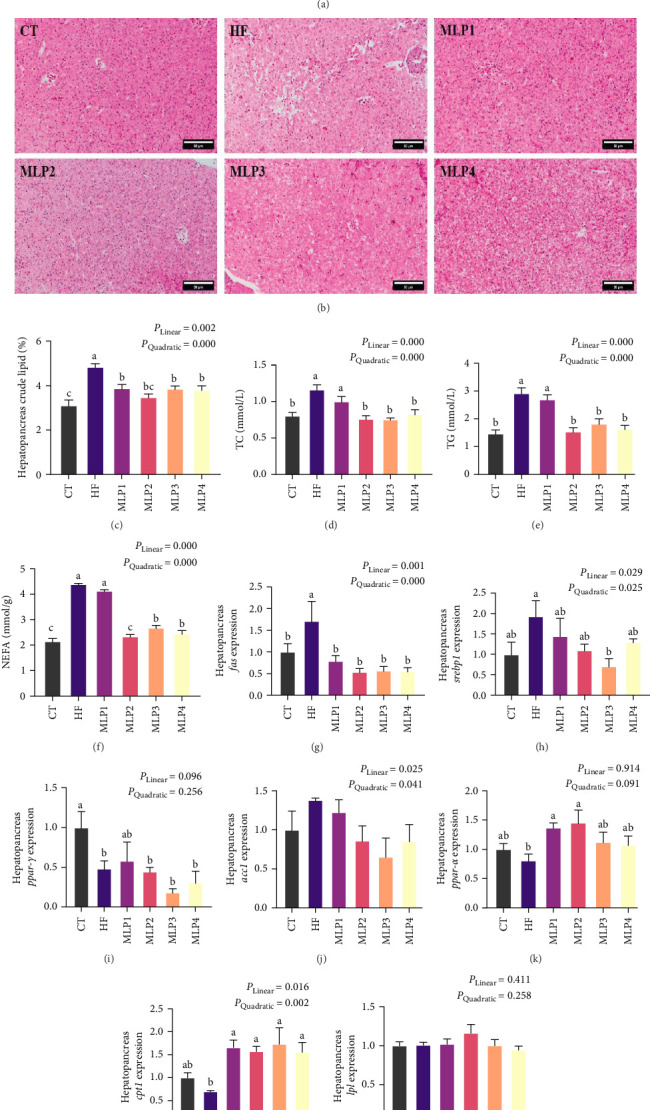
Effects of MLP supplementation in HFDs on hepatopancreas lipid metabolism of common carp (*n* = 3). (A) Anatomic diagram of hepatopancreas. (B) The observation of hepatopancreas (10x). (C–F) Biochemical indices of hepatic lipid metabolism: (C) crude lipid content of hepatopancreas, (D) TG (triglyceride) content, (E) TC (total cholesterol) content, and (F) NEFA (nonesterified fatty acid) content. (G–M) The gene expressions of lipid metabolism in hepatopancreas of common carp: (G) *fas* (*fatty acid synthase*) relative mRNA expression, (H) *srebp1* (*sterol regulatory element-binding protein 1*) relative mRNA expression, (I) *ppar-γ* (*peroxisome proliferator-activated receptor gamma*) relative mRNA expression, (J) *acc1* (*acetyl-coA carboxylase 1*) relative mRNA expression. (K) *ppar-α* (peroxisome proliferator-activated receptor alpha) relative mRNA expression, (L) *cpt-1* (*carnitine acyl transferase I*) relative mRNA expression, and (M) *lpl* (*lipoprotein lipase*) relative mRNA expression. CT, control group; HF, high lipid group; MLP1, the HFD supplemented with 0.6% MLP group; MLP2, the HFD supplemented with 1.2% MLP group; MLP3, the HFD supplemented with 1.8% MLP group; MLP4, the HFD supplemented with 2.4% MLP group. Values are means with SEM represented by vertical bars. Different superscript letters (a, b, c) indicate statistically significant differences among groups (*p* < 0.05). *p*-Values for the linear and quadratic relationships with dietary MLP levels were determined by orthogonal polynomial analysis.

Anatomic maps of the hepatopancreas revealed lipid accumulation in both the HF and MLP1 groups. This effect was more pronounced in the HF group (Figure 3A). H&E staining revealed that hepatocyte morphology in the CT group was well-defined, exhibiting minimal vacuolation and no significant pathological conditions. In contrast, the HF group displayed widened gaps between hepatocytes, dislocated nuclei, markedly increased lipid vacuolation, and disordered cellular arrangement. In the MLP1 and MLP2 groups, hepatocyte vacuolation was significantly reduced, cell boundaries were distinct, nuclei were centrally located within the hepatocytes, and the cells were tightly and regularly arranged. Compared to the HF group, the MLP3 group showed a decrease in hepatocyte vacuolation, with minimal nuclear migration observed. In the MLP4 group, hepatocytes were enlarged, showing small lipid droplets dispersed throughout the cytoplasm, with nuclei positioned centrally (Figure 3B).

The crude lipid content and the levels of TG, TC, and NEFA in the hepatopancreas of the HF group were higher than those in the CT group (*p* < 0.05). The crude lipid content in MLP groups was significantly lower than that in the HF group (*p* < 0.05). Furthermore, the levels of TG, TC, and NEFA in the hepatopancreas of fish fed diets with over 0.6% MLP were significantly lower than those in the HF group (*p* < 0.05). Moreover, the NEFA level in the MLP2 group was not significantly different from that in the CT group (*p* > 0.05) (Figure 3).

The mRNA expression of *fas*, *srebp1*, and *lpl* in the hepatopancreas of the HF group was upregulated compared to the CT group (*p* < 0.05). Concurrently, the mRNA expression of *fas*, *srebp1*, and *acc1* exhibited a downward trend with the addition of MLP. The expression of *fas* in the hepatopancreas of fish fed with MLP was significantly lower than that in the HF group (*p* < 0.05). The expression of *srebp1* in the hepatopancreas of the MLP3 group was significantly lower than that in the HF group (*p* < 0.05). The expression of *acc1* in the hepatopancreas of the MLP groups displayed a downward trend, although there was no significant difference compared to the HF group. The expression of *ppar-γ* in the hepatopancreas of the MLP groups did not differ significantly from that in the HF group. The lipolysis genes, including *ppar-α*, *cpt1*, and *lpl*, were measured. The gene expression of *cpt1* in the MLP groups and that of *ppar-α* in the MLP1 and MLP2 groups were significantly increased compared with the HF group (*p* < 0.05)(Figure 3).

**Figure 4:**
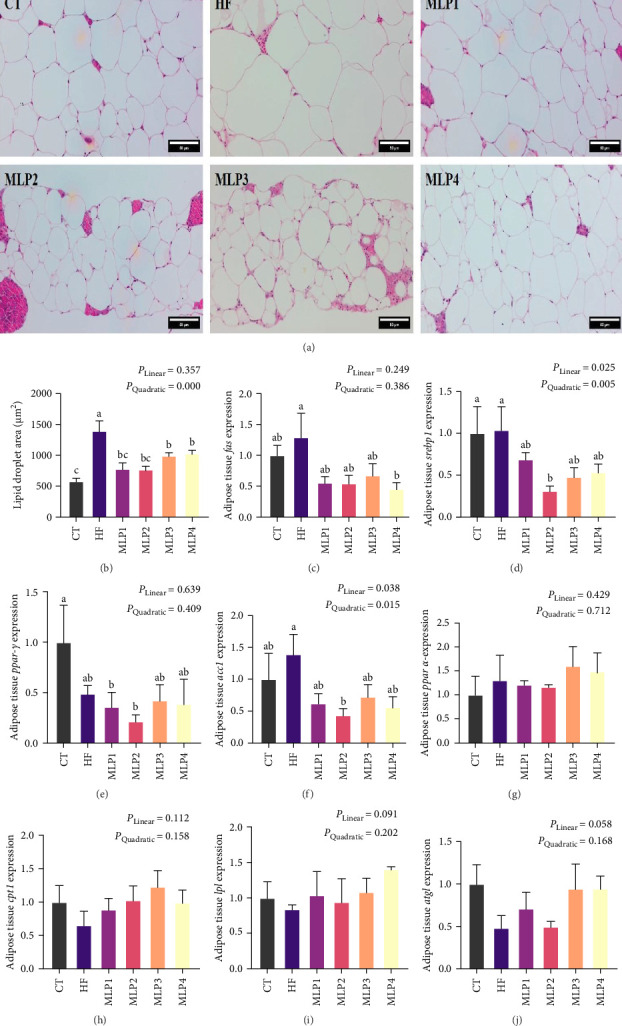
Effects of MLP supplementation in HFDs on adipose tissue lipid metabolism of common carp (*n* = 3). (A) The observation of adipose tissue of common carp (10x). (B) The adipocyte size statistics results. (C–J) The gene expressions of lipid metabolism in adipose tissue: (C) *fas* (*fatty acid synthase*) relative mRNA expression, (D) *srebp1* (*sterol regulatory element-binding protein 1*) relative mRNA expression, (E) *ppar-γ* (*peroxisome proliferator-activated receptor gamma*) relative mRNA expression, and (F) *acc1* (*acetyl-coA carboxylase 1*) relative mRNA expression, (G) *ppar-α* (peroxisome proliferator-activated receptor alpha) relative mRNA expression, (H) *cpt-1* (*carnitine acyl transferase I*) relative mRNA expression, (I) *lpl* (*lipoprotein lipase*) relative mRNA expression, and (J) *atgl* (*adipose triglyceride lipase*) relative mRNA expression. CT, control group; HF, high lipid group; MLP1, the HFD supplemented with 0.6% MLP group; MLP2, the HFD supplemented with 1.2% MLP group; MLP3, the HFD supplemented with 1.8% MLP group; MLP4, the HFD supplemented with 2.4% MLP group. Values are means with SEM represented by vertical bars. Different superscript letters (a, b, c) indicate statistically significant differences among groups (*p* < 0.05). *p*-Values for the linear and quadratic relationships with dietary MLP levels were determined by orthogonal polynomial analysis.

The effect of MLP supplementation in HFDs on the adipose tissue morphology of common carp was illustrated in Figure 4A. The adipocyte size in the HF group was significantly increased (*p* < 0.05). However, this effect was mitigated with the inclusion of MLP, as evidenced by significantly smaller adipocyte size in the MLP1, MLP2, MLP3, and MLP4 groups compared to the HF group (*p* < 0.05) (Figure 4B).

The effects of MLP added to HFDs on the gene expression of lipid metabolism in the adipose tissue of common carp were illustrated in Figure 4. The expression levels of lipid synthesis genes, including *fas*, *srebp1*, *ppar-γ*, and *acc1*, in the adipose tissue of the HF group did not differ significantly from those of the CT group. These lipid synthesis genes were downregulated in the adipose tissue of common carp after MLP supplementation. The expression of *fas* in the MLP4 group was significantly lower than that in the HF group (*p* < 0.05). The expression of *srebp1* and *acc1* in the MLP2 group was significantly downregulated compared to those in the HF group (*p* < 0.05). The expression of *ppar-γ* in the MLP2 group was significantly reduced compared to the CT group (*p* < 0.05). Conversely, the expression of lipolysis genes, including *ppar-α*, *cpt1*, *lpl*, and *atgl*, in the adipose tissue of common carp was upregulated after MLP supplementation, although no significant differences were observed among the various groups (*p* > 0.05).

### 3.8. Dietary MLP Requirements for Common Carp in HFDs

**Figure 5:**
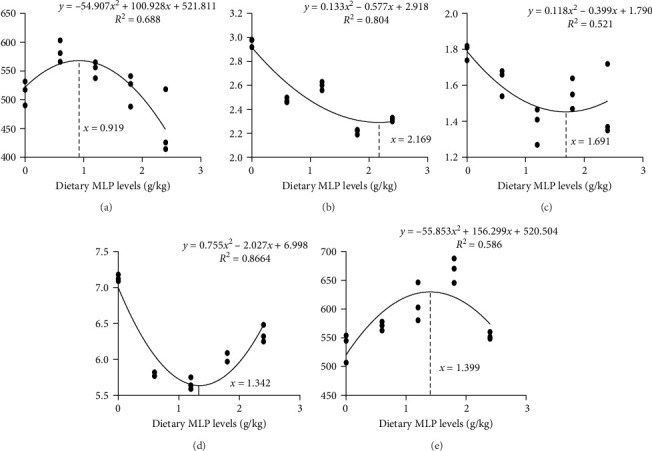
Optimal dietary MLP levels for common carp based on WGR (A), LDL level (B), TG level (C), MDA content (D), and villus height (E).

Based on WGR, TG level, LDL level, MDA content, and villus height, the dietary MLP levels for common carp fed HFDs were determined to be 0.9%, 1.69%, 2.17%, 1.34%, and 1.40%, respectively (Figure 5).

## 4. Discussion

### 4.1. MLP Addition in HFDs-Improved Fish Growth Performance

Lipids are essential nutrients that provide the energy necessary for the growth and development of aquatic animals. However, prolonged feeding of diets with elevated lipid levels can lead to issues such as reduced growth rates, excessive lipid accumulation, and diminished immune function in fish [31]. Our study indicated that fish growth and feed utilization were negatively affected by the excess of dietary lipids, which aligns with earlier studies performed on grass carp (*C. idella*) and black seabream (*Acanthopagrus schlegelii*) [32, 33]. The HFDs in our experiment resulted in a smaller decrease in body weight (10.38% reduction compared to control) of common carp, in contrast to the greater reductions observed in the studies by Yang et al. [34] and Du et al. [35], which reported decreases of 14.94% and 13.41%, respectively. This discrepancy may be attributed to the higher fat content in the experimental diets of the cited studies (11% and 15.92%, respectively), compared to the 10% fat content in our HFD. Studies have indicated that juvenile fish are particularly sensitive to changes in dietary nutrient composition [36, 37], and the fish in all three studies were of comparable size (~6 g). However, in our study, the addition of 0.6% to 1.2% MLP to HFDs significantly enhanced fish growth and feed utilization. Similarly, dietary supplementation with 0.5%–1% *Artemisia absinthium* significantly increased fish growth and feed efficiency in common carp [38]. Furthermore, rainbow trout (*Oncorhynchus mykiss*) fed an intermediate dose of *Artemisia dracunculus* also exhibited the highest growth [39].

### 4.2. MLP Addition in HFDs-Improved Fish Intestinal Health

Intestinal health is a crucial factor influencing the growth of aquatic animals. For instance, an increase in height or surface area of intestinal villi enhances the contact area between nutrients and the intestine [29]. Additionally, the enhancement of intestinal digestive enzyme activity can change how effectively fish break down and absorb nutrients from their diets [40]. Research has demonstrated that long-term feeding of HFDs, including some species including juvenile black seabream (*A. schlegelii*), juvenile rice field eels (*Monopterus albus*), zebrafish, and tilapia (*Oreochromis niloticus*), could lead to damage in intestinal structure or a reduction in digestive enzyme activity, thereby inhibiting the growth of these HF aquatic animals [41–44]. This study also reflects the negative effects of HFDs on the intestinal health of aquatic animals. In our experiment, the addition of MLP to HFDs resulted in a significant increase in intestinal villi height in the MLP1, MLP2, and MLP3 groups compared to the HF group. Moreover, the activities of intestinal digestive enzymes in common carp fed diets containing MLP were increased to varying degrees compared with those in the HF group. Therefore, we speculated that an appropriate level of MLP in HFDs could enhance the growth and feed utilization of aquatic animals by improving intestinal health. The beneficial effects of MLP supplementation on fish growth are dose-dependent, and exceeding optimal doses may negate these benefits. This finding aligns with the results of a meta-analysis regarding the impact of plants on aquatic animals [45].

In this experiment, mugwort leaf demonstrated the ability to enhance disease resistance in fish. The study found that the addition of MLP resulted in an upregulation of *il-10* in the MLP groups. Notably, *il-10* expression in the MLP2 group was significantly increased compared to the HF group, while the expression of *il-1β* and *tnf-α* was down-regulated compared to the HF group. These results indicated that the inclusion of MLP could alleviate intestinal inflammation caused by HFDs in common carp. Numerous studies have shown that mugwort leaf, which contains alkaloids, flavonoids, and various other compounds, can act as immune system stimulants that regulate the secretion of cytokines or antibodies, thereby enhancing the body's resistance to disease [46]. For instance, crude extracts of mugwort leaf have been shown to improve the immune capacity of mice suffering from dermatitis [47]. Furthermore, incorporating 1 g/kg of water extract from mugwort leaf into the diets of broilers daily can significantly enhance their immune capacity [48]. Additionally, mugwort leaf essential oil has been found to significantly inhibit levels of *tnf-α* and *il-6* while clearing ROS to suppress the inflammatory response by increasing the expression of *ppar-γ* [49]. A concentration of 0.5%–1.0% *A. absinthium* has been shown to significantly improve endogenous immunity in juvenile common carp [38]. Similarly, a study on zebrafish has demonstrated that essential oil extracted from *Artemisia vulgaris* could promote the development of intestinal structure and regulate the gene expression of the *myd88/Traf6/nf-κb* signaling pathway, thereby enhancing fish immunity [50].

### 4.3. MLP Addition in HFDs-Regulated Lipid Metabolism of Fish

The use of HFDs in intensive aquaculture of aquatic animals often leads to excessive lipid deposition in fish, resulting in lipid metabolism disorders that adversely affect their health [51]. Lipid metabolism in fish encompasses not only serum lipid metabolism but also tissue lipid metabolism, such as the liver and adipose tissue [52]. Blood serves as a critical medium for nutrient transport in aquatic animals, with serum containing a high concentration of metabolic substances that can be utilized to assess the nutritional and physiological status of fish during the feeding period [53]. The liver plays a pivotal role in lipid metabolism, participating in the synthesis, decomposition, and transport of lipid substances, while adipose tissue is essential for lipid storage [54, 55]. In this experiment, the serum ALT, AST, TC, and LDL levels in the HF group were significantly higher than those in the CT group. After feeding fish with diets containing MLP, the serum ALT, AST, and LDL in fish decreased, and both serum TG and TC levels exhibited a downward trend, with the TG level in the MLP2 group being significantly lower than that in the HF group. Additionally, the TC level in the MLP3 group was significantly lower than that in the HF group. These results indicated that HFDs could lead to abnormal lipid metabolism and liver health deterioration, while the inclusion of MLP had a beneficial regulatory effect on the lipid synthesis and decomposition in fish. Furthermore, the occurrence of oxidative stress is a significant contributor to liver metabolic disorders and liver injury [56, 57]. This study found that the addition of MLP significantly enhanced the activity of serum antioxidant enzymes and reduced serum MDA levels, suggesting that MLP may ameliorate oxidative stress induced by HFDs by modulating the antioxidant enzyme system, thereby mitigating liver damage in fish.

The degree of hepatocyte vacuolation of the MLP groups was reduced, with clear hepatocyte boundaries and centrally located nuclei. Additionally, the adipocyte size was significantly smaller, providing visual evidence for the effective regulation of MLP on lipid metabolism in the hepatopancreas and adipose tissue of carp fed HFDs. From a genetic perspective, the addition of MLP to the diets resulted in a downward trend in the expression of lipid synthesis genes, including *srebp1*, *fas*, and *acc1*, in both the hepatopancreas and adipose tissue of the MLP groups compared to the HF group. As a key transcription factor regulating lipid synthesis, *srebp1* influences the uptake of glycerol by hepatocytes and is an important factor in the absorption and synthesis of FAs and TC [58]. The *acc* and *fas* genes are critical rate-limiting enzymes in the FA synthesis process; their downregulation inhibits the synthesis of FAs, TC, and TG, thereby leading to a significant decrease in TG, TC, and NEFA in the liver [59]. Therefore, MLP may ameliorate excessive lipid accumulation caused by HFDs feeding in common carp primarily by inhibiting lipid synthesis. Similarly, findings regarding the effects of MLP-related products on lipid metabolism in other animals align with our results. For instance, Seomaeyakssuk (*A. argyi*) vinegar demonstrated a positive regulatory effect on lipid metabolism in mice subjected to the high-carbohydrate and HFD [60]. Additionally, incorporating 0.05% *Artemisia annua* into the diet of largemouth bass (*Micropterus salmoides*) significantly reduced fat accumulation in their livers [61]. Furthermore, zebrafish liver injury induced by anhydrous alcohol showed considerable recovery after treatment with mugwort essential oil [24]. *Artemisia folium polysaccharide* significantly decreased the levels of TC and LDL in plasma, as well as the expression of *fas* in the liver of ovariectomized mice, thus enhancing the lipid metabolism capacity [62].

## 5. Conclusion

Our study indicated that common carp fed HFDs supplemented with an appropriate dose of MLP exhibited improved growth performance, regulated lipid metabolism, and enhanced intestinal health. According to the regression analysis of the above indexes, the appropriate dosage range for MLP supplementation was 0.92% to 2.17%. This study reveals the potential function of MLP in regulating lipid metabolism and intestinal health of common carp after HFD feeding, which can provide new ideas for the green and healthy development of the aquaculture industry.

## Figures and Tables

**Table 1:** Dietary formulation and nutritional composition (dry matter basis, g/kg).

Ingredients	CT^a^	HF^b^	MLP1^c^	MLP2^d^	MLP3^e^	MLP4^f^
Fish meal	100.00	100.00	100.00	100.00	100.00	100.00
Rapeseed meal	220.00	220.00	220.00	220.00	220.00	220.00
Cottonseed meal	190.00	190.00	190.00	190.00	190.00	190.00
Soybean meal	140.00	140.00	140.00	140.00	140.00	140.00
Rice bran	100.00	100.00	100.00	100.00	100.00	100.00
Wheat flour	85.00	85.00	85.00	85.00	85.00	85.00
Wheat bran	45.00	45.00	39.00	33.00	27.00	21.00
MLP^g^	0.00	0.00	6.00	12.00	18.00	24.00
Microcrystalline cellulose	62.00	10.00	10.00	10.00	10.00	10.00
Soybean oil	22.00	74.00	74.00	74.00	74.00	74.00
Ca(H_2_PO_4_)_2_	20.00	20.00	20.00	20.00	20.00	20.00
Premix^h^	10.00	10.00	10.00	10.00	10.00	10.00
Choline chloride	5.00	5.00	5.00	5.00	5.00	5.00
Ethoxyquin	1.00	1.00	1.00	1.00	1.00	1.00
Total	1000.00	1000.00	1000.00	1000.00	1000.00	1000.00
Nutritional composition (%)						
Moisture	6.47	7.08	6.62	6.82	6.23	6.79
Crude protein	36.19	36.16	36.26	36.27	36.23	36.21
Crude lipid	4.76	10.04	10.14	10.16	10.08	10.12
Crude ash	8.52	8.66	8.24	8.28	8.37	8.48

^a^CT = Control group.

^b^HF = High lipid group.

^c^MLP1 = The HFD supplemented with 0.6% MLP group.

^d^MLP2 = The HFD supplemented with 1.2% MLP group.

^e^MLP3 = The HFD supplemented with 1.8% MLP group.

^f^MLP4 = The HFD supplemented with 2.4% MLP group.

^g^MLP contained 23.23% crude protein and 3.45% crude lipid.

^h^Premix: 1 % vitamin, 1 % mineral, and 1 % limestone carrier; ingredients including/1 kg: vitamin A 4000 IU; vitamin D3 800 IU; vitamin E 50 IU; vitamin B1 2.5 mg; vitamin B2 9 mg; vitamin B6 10 mg; vitamin C 250 mg; nicotinic acid 40 mg; pantothenic acid calcium 30 mg; biotin100 μg; betaine 1000 mg; Fe 140 mg; Cu 2.5 mg; Zn 65 mg; Mn 19 mg; Mg 230 mg; Co 0.1 mg; I 0.25 mg; Se 0.2 mg.

**Table 2:** FA composition of experimental diets (%).

FAs	CT	HF	MLP1	MLP2	MLP3	MLP4
C14:0	0.64	0.36	0.34	0.36	0.34	0.36
C16:0	14.18	12.13	11.89	12.06	12.11	12.06
C18:0	21.97	13.69	17.51	15.98	17.60	16.03
∑ SFA	36.78	26.18	29.74	28.40	30.05	28.45
C16:1n-7	1.61	0.97	0.99	0.98	0.99	1.03
C18:1n-9	10.77	16.52	13.57	15.01	13.71	15.54
C20:1n-9	0.33	0.36	0.39	0.43	0.39	0.36
∑ MUFA	12.70	17.85	14.95	16.43	15.09	16.93
C18:2n-6	43.47	48.99	48.68	48.53	48.28	47.97
C18:3n-6	0.11	0.05	0.03	0.03	0.03	0.03
C20:4n-6	0.20	0.12	0.11	0.13	0.15	0.17
∑ n-6PUFA	43.78	49.15	48.82	48.69	48.45	48.17
C18:3n-3	5.28	6.05	5.73	5.72	5.63	5.61
C20:5n-3	0.62	0.30	0.31	0.31	0.30	0.32
C22:6n-3	0.84	0.46	0.45	0.45	0.47	0.53
∑ n-3PUFA	6.74	6.82	6.49	6.49	6.41	6.45
n-3/n-6PUFA	0.15	0.14	0.13	0.13	0.13	0.13

*Note*: CT, control group; HF, high lipid group; MLP1, the HFD supplemented with 0.6% MLP group; MLP2, the HFD supplemented with 1.2% MLP group; MLP3, the HFD supplemented with 1.8% MLP group; MLP4, the HFD supplemented with 2.4% MLP group.

Abbreviations: MUFA, monounsaturated fatty acid; PUFA, polyunsaturated fatty acid; SFA, saturated fatty acids.

**Table 3:** Main bioactive components of MLP screened by UHPLC-MS/MS (positive ion mode).

Identification	Formula	Adduct methods	*m*/*z*	Retention time (min)
Jaccesidin	C_17_H_14_O_7_	[M+H]+	331.081	8.88
Eupatilin	C_18_H_16_O_7_	[M+H]+	345.097	9.61
Luteolin	C_15_H_10_O_6_	[M+H]+	287.055	8.60
Apigenin	C_15_H_10_O_5_	[M+H]+	271.060	8.46
Nepetin	C_16_H_12_O_7_	[M+H]+	317.066	8.71
Chrysoeriol	C_16_H_12_O_6_	[M+H]+	301.071	8.53
Acacetin	C_16_H_12_O_5_	[M+H]+	285.076	10.82
Ladanein	C_17_H_14_O_6_	[M+H]+	315.086	10.42
Bonanzin	C_18_H_16_O_8_	[M+H]+	361.092	8.77
Diosmin	C_28_H_32_O_15_	[M+H]+	609.181	6.21
5,6,4′-trihydroxy-7,3′-dimethoxyflavone	C_17_H_14_O_8_	[M+H]+	347.076	8.73
Casticin	C_19_H_18_O_8_	[M+H]+	375.107	10.04
Artemetin	C_20_H_20_O_8_	[M+H]+	389.123	11.08
Luteolin-7-glucuronic acid	C_21_H_18_O_12_	[M+H]+	463.087	5.95
Eupatilin 7-O-β-D-glucopyranoside	C_24_H_26_O_12_	[M+H]+	507.150	10.92
Quercetin	C_15_H_10_O_7_	[M+H]+	303.050	7.84
Kaempferol	C_15_H_10_O_6_	[M+H]+	287.055	8.60
Eupatolitin	C_17_H_14_O_8_	[M+H]+	347.076	8.73
Apicin	C_21_H_20_O_10_	[M+H]+	433.113	6.52
Isoquercetin	C_21_H_20_O_12_	[M+H]+	465.103	6.10
Rutin	C_27_H_30_O_16_	[M+H]+	611.161	5.59
Naringenin	C_15_H_12_O_5_	[M+H]+	273.076	8.58
Homoeriodictyol	C_16_H_14_O_6_	[M+H]+	303.086	8.69
5,7,3′,4′-tetrahydroxyflavone	C_15_H_12_O_6_	[M+H]+	289.071	7.83
Protocatechuic acid	C_7_H_6_O_4_	[M+H]+	155.034	6.42
Salicylic alcohol-dihexoside	C_19_H_28_O_13_	[M+H]+	465.160	5.71
Neochlorogenic acids	C_16_H_18_O_9_	[M+H]+	355.102	4.55
Trans-o-coumaric acid	C_9_H_8_O_3_	[M+H]+	165.055	7.64
Caffeic acid	C_9_H_8_O_4_	[M+H]+	181.050	5.20
5-feruloylquinic acid	C_17_H_20_O_9_	[M+H]+	369.118	5.36
1,3-dicaffeoylquinic acid	C_25_H_24_O_12_	[M+H]+	517.134	6.61
3-caffeoyl-5-feruloyl-quinic acid	C_26_H_26_O_12_	[M+H]+	531.150	7.94
3,4,5-tricaffeoylquinic acid	C_34_H_30_O_15_	[M+H]+	679.166	7.54
Hydroxycinnamic acid-O-sulfate	C_9_H_8_O_7_S	[M+H]+	261.006	7.14
Azelaic acid	C_9_H_16_O_4_	[M+H]+	189.112	6.94
9,12,13-trihydroxy octadecenoic acid	C_18_H_34_O_5_	[M+H]+	331.248	8.15
Limonene	C_10_H_16_	[M+H]+	137.132	10.47
Camphor	C_10_H_16_O	[M+H]+	153.127	10.65
Caryophyllene	C_15_H_24_	[M+H]+	205.195	13.62
Geranyl acetate	C_12_H_20_O_2_	[M+H]+	197.154	7.35
Resveratrol	C_14_H_12_O_3_	[M+H]+	229.086	7.42
Umbelliferone	C_9_H_6_O_3_	[M+H]+	163.039	8.85

**Table 4:** Effects of MLP in HFDs on growth performance and morphology of common carp (*n* = 3).

Parameters	Groups						Polynomial contrasts (*p*-value)	
	CT	HF	MLP1	MLP2	MLP3	MLP4	Linear	Quadratic
IBW (g)	6.01 ± 0.01	6.00 ± 0.02	6.01 ± 0.01	6.02 ± 0.01	6.03 ± 0.01	6.01 ± 0.01	0.239	0.211
FBW (g)	41.03 ± 0.64^a^	36.77 ± 0.68^bc^	41.08 ± 0.67^a^	39.32 ± 0.45^a^	37.30 ± 0.98^b^	33.24 ± 1.97^c^	0.057	0.001
WGR (%)	582.70 ± 9.97^a^	513.14 ± 12.13^b^	583.52 ± 10.74^a^	553.14 ± 8.16^a^	518.95 ± 15.87^b^	452.87 ± 32.92^c^	0.050	0.001
SGR (%)	3.31 ± 0.03^a^	3.13 ± 0.04^b^	3.31 ± 0.03^a^	3.23 ± 0.03^ab^	3.14 ± 0.05^ab^	2.94 ± 0.10^c^	0.774	0.000
FCR (%)	1.21 ± 0.02^a^	1.45 ± 0.04^b^	1.27 ± 0.03^a^	1.31 ± 0.02^ab^	1.29 ± 0.04^b^	1.46 ± 0.09^a^	0.084	0.065
SR (%)	100.00 ± 0.00	98.89 ± 1.11	100.00 ± 0.00	100.00 ± 0.00	100.00 ± 0.00	100.00 ± 0.00	0.165	0.185
VSI (%)	5.67 ± 0.29	5.73 ± 0.32	5.99 ± 0.49	5.59 ± 0.07	6.56 ± 0.28	6.42 ± 0.46	0.544	0.510
HSI (%)	1.27 ± 0.04	1.31 ± 0.05	1.13 ± 0.03	1.25 ± 0.05	1.37 ± 0.09	1.31 ± 0.02	0.297	0.400
SI (%)	0.23 ± 0.01^ab^	0.17 ± 0.01^b^	0.23 ± 0.02^ab^	0.25 ± 0.03^ab^	0.23 ± 0.01^ab^	0.30 ± 0.03^a^	0.006	0.029
KI (%)	0.42 ± 0.01	0.44 ± 0.01	0.42 ± 0.02	0.38 ± 0.05	0.41 ± 0.04	0.39 ± 0.02	0.288	0.482
IFI (%)	0.32 ± 0.01	0.41 ± 0.06	0.38 ± 0.03	0.33 ± 0.02	0.43 ± 0.04	0.36 ± 0.02	0.199	0.199
RIL (%)	1.55 ± 0.10	1.36 ± 0.02	1.40 ± 0.05	1.40 ± 0.04	1.50 ± 0.05	1.56 ± 0.13	0.027	0.082
CF (g/cm^3^)	2.56 ± 0.04	2.55 ± 0.05	2.53 ± 0.05	2.56 ± 0.01	2.56 ± 0.07	2.46 ± 0.04	0.506	0.543

*Note:* Data are presented as mean ± SEM. Different superscript letters (a, b, c) indicate statistically significant differences among groups (*p* < 0.05); *p*-values for the linear and quadratic relationships with dietary MLP levels were determined by orthogonal polynomial analysis. CT, control group; MLP1, the HFD supplemented with 0.6% MLP group; MLP2, the HFD supplemented with 1.2% MLP group; MLP3, the HFD supplemented with 1.8% MLP group; MLP4, the HFD supplemented with 2.4% MLP group.

Abbreviations: CF, condition factor; FBW, final body weight; FCR, feed conversion ratio; HF, high lipid group; HSI, hepatosomatic index; IBW, initial body weight; IFI, intraperitoneal fat index; KI, kidney index; RIL, relative intestine length; SGR, specific growth rate; SI, spleen index; SR, survival rate; VSI, viscerosomatic index; WGR, weight gain rate.

**Table 5:** Effects of MLP in HFDs on body composition of common carp (wet weight, %, *n* = 3).

Parameters	Groups						Polynomial contrasts (*p*-value)	
	CT	HF	MLP1	MLP2	MLP3	MLP4	Linear	Quadratic
Whole-body								
Moisture	76.15 ± 0.43^a^	73.69 ± 0.43^b^	72.46 ± 0.70^b^	73.23 ± 0.20^b^	73.34 ± 0.54^b^	74.96 ± 0.43^a^	0.054	0.005
Crude protein	14.91 ± 0.44	14.09 ± 0.30	14.77 ± 0.63	14.01 ± 0.26	14.57 ± 0.20	14.21 ± 0.25	0.975	0.865
Crude lipid	4.33 ± 0.22^c^	8.00 ± 0.20^a^	6.66 ± 0.14^b^	6.78 ± 0.26^b^	6.58 ± 0.15^b^	6.42 ± 0.61^b^	0.007	0.009
Crude ash	2.82 ± 0.08	2.90 ± 0.08	3.08 ± 0.20	2.97 ± 0.38	2.92 ± 0.20	2.93 ± 0.02	0.892	0.923
Muscle								
Moisture	78.93 ± 0.09	78.50 ± 0.35	78.76 ± 0.40	78.53 ± 0.08	78.51 ± 0.15	79.43 ± 0.29	0.079	0.070
Crude protein	17.13 ± 0.10	17.07 ± 0.79	18.17 ± 0.28	17.72 ± 0.51	17.58 ± 0.45	16.58 ± 0.56	0.399	0.118
Crude lipid	0.39 ± 0.03^c^	1.03 ± 0.17^ab^	1.01 ± 0.11^b^	1.25 ± 0.15^ab^	1.44 ± 0.11^a^	0.85 ± 0.10^b^	0.828	0.082
Crude ash	1.29 ± 0.03	1.28 ± 0.03	1.24 ± 0.04	1.25 ± 0.01	1.26 ± 0.03	1.21 ± 0.09	0.892	0.923

*Note:* Data are presented as mean ± SEM. Different superscript letters (a, b, c) indicate statistically significant differences among groups (*p* < 0.05); *p*-values for the linear and quadratic relationships with dietary MLP levels were determined by orthogonal polynomial analysis. CT, control group; HF, high lipid group; MLP1, the HFD supplemented with 0.6% MLP group; MLP2, the HFD supplemented with 1.2% MLP group; MLP3, the HFD supplemented with 1.8% MLP group; MLP4, the HFD supplemented with 2.4% MLP group.

**Table 6:** Effects of MLP in HFDs on whole FAs composition of common carp (%, *n* = 3).

Parameters	Groups						Polynomial contrasts (*p*-value)	
	CT	HF	MLP1	MLP2	MLP3	MLP4	Linear	Quadratic
C14:0	0.85 ± 0.02^a^	0.50 ± 0.00^b^	0.50 ± 0.00^b^	0.52 ± 0.00^b^	0.46 ± 0.00^c^	0.45 ± 0.01^c^	0.000	0.000
C16:0	17.50 ± 0.39^a^	13.27 ± 0.16^b^	12.77 ± 0.15^bc^	12.88 ± 0.11^bc^	12.29 ± 0.05^c^	12.17 ± 0.19^c^	0.000	0.000
C18:0	24.06 ± 1.69	22.45 ± 0.69	23.44 ± 0.43	24.27 ± 0.36	23.21 ± 0.71	22.20 ± 0.62	0.715	0.030
SFA	42.41 ± 1.49^a^	36.22 ± 0.64^bc^	36.72 ± 0.32^bc^	37.67 ± 0.41^b^	35.95 ± 0.70^bc^	34.82 ± 0.60^c^	0.081	0.006
C16:1	2.12 ± 0.15^a^	1.65 ± 0.06^b^	1.89 ± 0.12^ab^	2.21 ± 0.07^a^	2.25 ± 0.13^a^	2.29 ± 0.11^a^	0.000	0.000
C18:1	15.19 ± 1.00^a^	11.25 ± 0.38^b^	11.73 ± 0.22^b^	11.30 ± 0.27^b^	11.36 ± 0.39^b^	11.78 ± 0.52^b^	0.557	0.818
C20:1	1.54 ± 0.05^a^	0.92 ± 0.03^b^	0.98 ± 0.01^b^	0.95 ± 0.03^b^	0.91 ± 0.06^b^	0.92 ± 0.02^b^	0.449	0.526
MUFA	18.84 ± 0.96^a^	13.82 ± 0.35^b^	14.60 ± 0.14^b^	14.47 ± 0.33^b^	14.51 ± 0.43^b^	14.98 ± 0.62^b^	0.083	0.219
C18:2n-6	31.48 ± 0.58^b^	42.11 ± 0.42^a^	41.55 ± 0.22^a^	40.85 ± 0.40^a^	42.01 ± 0.58^a^	42.68 ± 0.36^a^	0.279	0.018
C18:3n-6	0.44 ± 0.05^c^	0.57 ± 0.04^bc^	0.64 ± 0.02^b^	0.55 ± 0.02^bc^	0.79 ± 0.06^a^	0.61 ± 0.03^b^	0.143	0.245
C20:4n-6	1.72 ± 0.16^a^	1.09 ± 0.05^b^	0.99 ± 0.02^b^	1.06 ± 0.04^b^	1.04 ± 0.04^b^	1.15 ± 0.11^b^	0.333	0.223
n-6PUFA	33.64 ± 0.59^b^	43.77 ± 0.45^a^	43.18 ± 0.24^a^	42.46 ± 0.42^a^	43.84 ± 0.60^a^	44.44 ± 0.48^a^	0.212	0.021
C18:3n-3	3.29 ± 0.08^c^	4.90 ± 0.04^a^	4.40 ± 0.02^b^	4.30 ± 0.06^b^	4.46 ± 0.05^b^	4.35 ± 0.02^b^	0.000	0.000
C20:5n-3	0.22 ± 0.01^a^	0.18 ± 0.01^b^	0.16 ± 0.00^b^	0.17 ± 0.01^b^	0.16 ± 0.00^b^	0.16 ± 0.01^b^	0.037	0.030
C22:6n-3	1.59 ± 0.09^a^	1.12 ± 0.07^bc^	0.94 ± 0.03^c^	0.94 ± 0.09^c^	1.07 ± 0.06^bc^	1.24 ± 0.11^b^	0.169	0.008
n-3PUFA	5.10 ± 0.11^c^	6.20 ± 0.08^a^	5.50 ± 0.02^b^	5.40 ± 0.08^c^	5.69 ± 0.08^b^	5.75 ± 0.13^b^	0.110	0.000
n-3/n-6PUFA	0.15 ± 0.00^a^	0.14 ± 0.00^b^	0.13 ± 0.00^c^	0.13 ± 0.00^c^	0.13 ± 0.00^c^	0.13 ± 0.00^c^	0.004	0.000

*Note:* Data are presented as mean ± SEM. Different superscript letters (a, b, c) indicate statistically significant differences among groups (*p* < 0.05); *p*-values for the linear and quadratic relationships with dietary MLP levels were determined by orthogonal polynomial analysis. CT, control group; HF, high lipid group; MLP1, the HFD supplemented with 0.6% MLP group; MLP2, the HFD supplemented with 1.2% MLP group; MLP3, the HFD supplemented with 1.8% MLP group; MLP4, the HFD supplemented with 2.4% MLP group.

Abbreviations: MUFA, monounsaturated fatty acids; PUFA, polyunsaturated fatty acids; SFA, saturated fatty acids.

**Table 7:** Effects of MLP in HFDs on muscle FAs composition of common carp (%, *n* = 3).

Parameters	Groups						Polynomial contrasts (*p*-value)	
	CT	HF	MLP1	MLP2	MLP3	MLP4	Linear	Quadratic
C14:0	0.85 ± 0.02^a^	0.49 ± 0.01^b^	0.50 ± 0.00^b^	0.52 ± 0.00^b^	0.46 ± 0.00^c^	0.45 ± 0.01^c^	0.001	0.000
C16:0	17.50 ± 0.39^a^	13.18 ± 0.15^b^	12.77 ± 0.15^bc^	12.88 ± 0.11^bc^	12.29 ± 0.05^c^	12.17 ± 0.19^c^	0.000	0.000
C18:0	24.06 ± 1.69	22.29 ± 0.67	23.44 ± 0.43	24.27 ± 0.36	23.21 ± 0.71	22.20 ± 0.62	0.839	0.021
SFA	42.41 ± 1.49^a^	35.96 ± 0.59^bc^	36.72 ± 0.32^bc^	37.67 ± 0.41^b^	35.95 ± 0.70^bc^	34.82 ± 0.60^c^	0.135	0.004
C16:1	2.12 ± 0.15^a^	1.64 ± 0.07^b^	1.89 ± 0.12^ab^	2.21 ± 0.07^a^	2.25 ± 0.13^a^	2.29 ± 0.11^a^	0.000	0.000
C18:1	15.19 ± 1.00^a^	11.17 ± 0.41^b^	11.73 ± 0.22^b^	11.30 ± 0.27^b^	11.36 ± 0.39^b^	11.78 ± 0.52^b^	0.483	0.778
C20:1	1.54 ± 0.05^a^	0.92 ± 0.03^b^	0.98 ± 0.01^b^	0.95 ± 0.03^b^	0.91 ± 0.06^b^	0.92 ± 0.02^b^	0.524	0.512
MUFA	18.84 ± 0.96^a^	13.72 ± 0.39^b^	14.60 ± 0.14^b^	14.47 ± 0.33^b^	14..51 ± 0.43^b^	14.98 ± 0.62^b^	0.068	0.178
C18:2n-6	31.48 ± 0.58^c^	41.82 ± 0.53^ab^	41.55 ± 0.22^ab^	40.85 ± 0.40^b^	42.01 ± 0.58^ab^	42.68 ± 0.36^a^	0.279	0.018
C18:3n-6	0.44 ± 0.05^c^	0.57 ± 0.04^bc^	0.64 ± 0.02^b^	0.55 ± 0.02^bc^	0.79 ± 0.06^a^	0.61 ± 0.03^b^	0.143	0.245
C20:4n-6	1.72 ± 0.16^a^	1.09 ± 0.05^b^	0.99 ± 0.02^b^	1.06 ± 0.04^b^	1.04 ± 0.04^b^	1.15 ± 0.11^b^	0.333	0.223
n-6PUFA	33.64 ± 0.59^c^	43.77 ± 0.45^ab^	43.18 ± 0.24^ab^	42.46 ± 0.42^b^	43.84 ± 0.60^ab^	44.44 ± 0.48^a^	0.212	0.021
C18:3n-3	3.29 ± 0.08^c^	4.87 ± 0.06^a^	4.40 ± 0.02^b^	4.30 ± 0.06^b^	4.46 ± 0.05^b^	4.35 ± 0.02^b^	0.000	0.000
C20:5n-3	0.22 ± 0.01^a^	0.18 ± 0.01^b^	0.16 ± 0.00^b^	0.17 ± 0.01^b^	0.16 ± 0.00^b^	0.16 ± 0.01^b^	0.054	0.052
C22:6n-3	1.59 ± 0.09^a^	1.11 ± 0.07^bc^	0.94 ± 0.03^c^	0.94 ± 0.09^c^	1.07 ± 0.06^bc^	1.24 ± 0.11^b^	0.155	0.009
n-3PUFA	5.10 ± 0.11^c^	6.16 ± 0.11^a^	5.50 ± 0.02^b^	5.40 ± 0.08^c^	5.69 ± 0.08^b^	5.75 ± 0.13^b^	0.156	0.000
n-3/n-6PUFA	0.15 ± 0.00^a^	0.14 ± 0.00^b^	0.13 ± 0.00^c^	0.13 ± 0.00^c^	0.13 ± 0.00^c^	0.13 ± 0.00^c^	0.004	0.000

*Note:* Data are presented as mean ± SEM. Different superscript letters (a, b, c) indicate statistically significant differences among groups (*p* < 0.05); *p*-values for the linear and quadratic relationships with dietary MLP levels were determined by orthogonal polynomial analysis. CT, control group; HF, high lipid group; MLP1, the HFD supplemented with 0.6% MLP group; MLP2, the HFD supplemented with 1.2% MLP group; MLP3, the HFD supplemented with 1.8% MLP group; MLP4, the HFD supplemented with 2.4% MLP group.

Abbreviations: MUFA, monounsaturated fatty acids; PUFA, polyunsaturated fatty acids; SFA, saturated fatty acids.

**Table 8:** Effects of MLP in HFDs on hepatopancreas FAs composition of common carp (%, *n* = 3).

Parameters	Groups						Polynomial contrasts (*p*-value)	
	CT	HF	MLP1	MLP2	MLP3	MLP4	Linear	Quadratic
C14:0	065 ± 0.01^a^	0.48 ± 0.00^b^	0.41 ± 0.00^c^	0.43 ± 0.01^c^	0.43 ± 0.00^c^	0.43 ± 0.00^c^	0.020	0.002
C16:0	21.21 ± 0.41^a^	16.57 ± 0.05^c^	16.72 ± 0.12^c^	17.54 ± 0.07^b^	15.08 ± 0.07^d^	15.59 ± 0.14^d^	0.008	0.006
C18:0	10.92 ± 0.26^b^	11.33 ± 0.68^b^	10.50 ± 0.61^bc^	9.05 ± 0.27^c^	13.47 ± 0.41^a^	10.33 ± 0.30^bc^	0.732	0.884
SFA	32.78 ± 0.18^a^	28.38 ± 0.68^bc^	27.64 ± 0.66^bc^	27.02 ± 0.22^bc^	28.99 ± 0.47^b^	26.35 ± 0.27^c^	0.195	0.392
C16:1	2.17 ± 0.01^a^	1.42 ± 0.01^b^	1.41 ± 0.02^b^	1.41 ± 0.01^b^	1.38 ± 0.01^b^	1.40 ± 0.02^b^	0.136	0.257
C18:1	24.70 ± 0.26^a^	19.25 ± 0.63^c^	21.4 ± 0.72^b^	22.73 ± 0.21^b^	19.66 ± 0.42^c^	22.28 ± 0.25^b^	0.102	0.140
C20:1	1.41 ± 0.02^a^	0.94 ± 0.00^c^	0.98 ± 0.02^b^	1.02 ± 0.01^b^	1.00 ± 0.01^b^	0.93 ± 0.01^c^	0.955	0.000
MUFA	28.27 ± 0.29^a^	21.60 ± 0.63^c^	23.79 ± 0.75^b^	25.15 ± 0.21^b^	22.04 ± 0.44^c^	24.61 ± 0.24^b^	0.113	0.140
C18:2n-6	21.04 ± 0.37^d^	35.21 ± 0.20^b^	32.34 ± 0.24^c^	32.69 ± 0.09^c^	36.59 ± 0.10^a^	36.12 ± 0.26^a^	0.032	0.001
C18:3n-6	0.62 ± 0.02^e^	1.13 ± 0.01^a^	1.13 ± 0.01^a^	0.99 ± 0.02^b^	0.78 ± 0.01^d^	0.86 ± 0.03^c^	0.000	0.000
C20:4n-6	6.43 ± 0.18^a^	3.90 ± 0.11^c^	4.74 ± 0.05^b^	4.34 ± 0.04^b^	3.32 ± 0.03^d^	3.65 ± 0.13^cd^	0.018	0.011
n-6PUFA	28.09 ± 0.19^c^	40.24 ± 0.10^a^	38.21 ± 0.18^b^	38.02 ± 0.05^b^	40.69 ± 0.09^a^	40.62 ± 0.23^a^	0.100	0.001
C18:3n-3	1.59 ± 0.05^d^	3.25 ± 0.04^ab^	2.74 ± 0.04^c^	2.79 ± 0.01^c^	3.35 ± 0.02^a^	3.17 ± 0.04^b^	0.285	0.020
C20:5n-3	0.37 ± 0.00^a^	0.32 ± 0.01^b^	0.33 ± 0.00^b^	0.33 ± 0.00^b^	0.25 ± 0.00^c^	0.24 ± 0.01^c^	0.000	0.000
C22:6n-3	8.90 ± 0.34^a^	6.20 ± 0.23^c^	7.28 ± 0.09^b^	6.69 ± 0.06^bc^	4.68 ± 0.03^d^	5.01 ± 0.48^d^	0.002	0.001
n-3PUFA	10.86 ± 0.30^a^	9.77 ± 0.19^b^	10.35 ± 0.05^ab^	9.80 ± 0.05^b^	8.28 ± 0.04^c^	8.42 ± 0.46^c^	0.000	0.000
n-3/n-6PUFA	0.39 ± 0.01^a^	0.24 ± 0.01^b^	0.27 ± 0.00^b^	0.26 ± 0.00^b^	0.20 ± 0.00^c^	0.21 ± 0.01^c^	0.001	0.000

*Note:* Data are presented as mean ± SEM. Different superscript letters (a, b, c) indicate statistically significant differences among groups (*p* < 0.05); *p*-values for the linear and quadratic relationships with dietary MLP levels were determined by orthogonal polynomial analysis. CT, control group; HF, high lipid group; MLP1, the HFD supplemented with 0.6% MLP group; MLP2, the HFD supplemented with 1.2% MLP group; MLP3, the HFD supplemented with 1.8% MLP group; MLP4, the HFD supplemented with 2.4% MLP group.

Abbreviations: MUFA, monounsaturated fatty acids; PUFA, polyunsaturated fatty acids; SFA, saturated fatty acids.

**Table 9:** Effects of MLP in HFDs on serum biochemical parameters of common carp (*n* = 3).

Parameters	Groups						Polynomial contrasts (*p*-value)	
	CT	HF	MLP1	MLP2	MLP3	MLP4	Linear	Quadratic
Serum lipid metabolism								
ALT (U/L)	2.27 ± 0.24^b^	3.40 ± 0.25^a^	2.22 ± 0.07^b^	1.59 ± 0.19^b^	2.20 ± 0.24^b^	1.97 ± 0.19^b^	0.001	0.000
AST (U/L)	4.25 ± 0.31^bc^	5.71 ± 0.30^a^	4.20 ± 0.18^bc^	3.82 ± 0.07^c^	4.44 ± 0.14^bc^	4.78 ± 0.26^b^	0.123	0.000
TG (mmol/L)	1.55 ± 0.14^ab^	1.79 ± 0.03^a^	1.62 ± 0.04^ab^	1.40 ± 0.05^b^	1.55 ± 0.06^ab^	1.49 ± 0.09^ab^	0.006	0.002
TC (mmol/L)	7.36 ± 0.31^b^	8.53 ± 0.37^a^	7.96 ± 0.17^ab^	7.69 ± 0.38^ab^	7.44 ± 0.32^b^	7.48 ± 0.05^ab^	0.006	0.013
GLU (mmol/L)	6.46 ± 0.10	6.79 ± 0.18	6.52 ± 0.13	6.61 ± 0.13	6.96 ± 0.06	6.51 ± 0.22	0.860	0.960
HDL-C (mmol/L)	12.86 ± 0.56	11.93 ± 0.58	11.66 ± 0.25	12.20 ± 0.58	11.29 ± 0.48	12.26 ± 0.38	0.971	0.815
LDL-C (mmol/L)	2.11 ± 0.12^c^	2.96 ± 0.11^a^	2.48 ± 0.05^b^	2.59 ± 0.12^b^	2.21 ± 0.10^c^	2.30 ± 0.05^bc^	0.000	0.000
Serum antioxidant capacity								
T-AOC (U/mL)	1.90 ± 0.18^bc^	1.62 ± 0.13^c^	1.56 ± 0.14^c^	4.24 ± 0.20^a^	2.01 ± 0.18^bc^	2.47 ± 0.23^b^	0..037	0.000
SOD (U/mL)	62.57 ± 1.91	63.52 ± 1.56	62.65 ± 1.08	62.65 ± 2.13	58.45 ± 2.19	59.46 ± 0.92	0.010	0.036
CAT (U/mL)	0.76 ± 0.15	1.23 ± 0.26	0.87 ± 0.19	1.15 ± 0.13	0.78 ± 0.12	0.87 ± 0.14	0..161	0.357
MDA (nmol/mL)	5.43 ± 0.20^b^	7.13 ± 0.34^a^	5.80 ± 0.25^b^	5.66 ± 0.41^b^	6.05 ± 0.31^b^	6.35 ± 0.32^ab^	0.350	0.009

*Note:* Data are presented as mean ± SEM. Different superscript letters (a, b, c) indicate statistically significant differences among groups (*p*  < 0.05); *p*-values for the linear and quadratic relationships with dietary MLP levels were determined by orthogonal polynomial analysis. CT, control group; HF, high lipid group; MLP1, the HFD supplemented with 0.6% MLP group; MLP2, the HFD supplemented with 1.2% MLP group; MLP3, the HFD supplemented with 1.8% MLP group; MLP4, the HFD supplemented with 2.4% MLP group.

## Data Availability

The datasets during the current study are available from the corresponding author upon request.

## Supporting Information

Supporting Information Table S1: RT-qPCR primers. Table S2: The calculation formulas of the growth, feed utilization, and morphological indexes of fish fed experimental diets.
