# Supplementary material for: Mugwort Leaf Powder (Artemisia argyi) Alleviates Lipid Metabolism Disorders and Intestinal Health of Common Carp (Cyprinus carpio) Fed High-Fat Diets
Source: Aquac Nutr. 2025 Nov 3;2025:3972704. doi: 10.1155/anu/3972704 (PMC12602034; doi:10.1155/anu/3972704)
Supplement: Supporting Information — Table S1: RT-qPCR primers. Table S2: The calculation formulas of the growth, feed utilization, and morphological indexes of fish fed experimental diets. [file 3972704.f1.doc]

**Table S1 RT-qPCR primers**

| **Gene** | **Forward (5′ → 3′)** | **Reverse (5′ → 3′)** | **Accession no** |
| --- | --- | --- | --- |
| ***fas*** | GACAGGCCGCTATTGCTATT | TGCCGTAAGCTGAGGAAATC | GQ466045.1 |
| ***acc 1*** | TTCACTGGCGTATGAGGATATC | TCCACCTGTATGGTTCTTTGG | XM 019096370.1 |
| ***srebp 1*** | CGCCTGCTTCACTTCACTACTC | GGACCAGTCCTCATCCACAAA | XM_019073316.1 |
| ***pparγ*** | GCAAGGCAGTGGAGGACAAGAAC | ACGCAACACAGCACCATAAGAGG | XM_042734045.1 |
| ***atgl*** | CACCAACACCTCCATTCAGTTCACA | ACTCTTCATCCTCCTCACCGTCAG | KY906167.1 |
| ***cpt 1*** | CAGATGGAAAGTGTTGCTAATGAC | TGTGTAGAAGTTGCTGTTGACCA | JQ361077.1 |
| ***pparα*** | TGGTGACATGGAGGTGCTGGAG | TCTGCTGCTGTGTTGTTACTCTGG | XM_042722106.1 |
| ***lpl*** | CCGCTCCATTCACCTGTTCAT | GCTGAGACACATGCCCTTATT | FJ716101.1 |
| ***il-10*** | CGCCAGCATAAAGAACTCGT | TGCCAAATACTGCTCGATGT | KX964678.1 |
| ***il-1β*** | TTACAGTAAGACCAGCCTGA | AGGCTCGTCACTTAGTTTGT | AJ245635 |
| ***nf-κb*** | AACCAAGAACCAGCCGTACAAGC | ACTGTGTATCCTCCGCTCCTGTAG | MN167531.1 |
| ***tnf-α*** | AGGTGATGGTGTCGAGGAGGAAG | AGACTTGTTGAGCGTGAAGCAGAC | XM_019088899.2 |
| ***18s*** | GAGACTCCGGCTTGCTAAAT | CAGACCTGTTATTGCTCCATCT | FJ710826.1 |

note: *f**as* = *fatty acid synthetase; acc1 = acetyl-coa ccarboxylase 1; srebp 1 = sterol regulatory element-binding protein 1; ppar**γ =* *proliferator-activated receptor γ; atgl = adipose triglyceride lipase; cpt 1 = carnitine palmitoyl transferase 1; ppar**α = proliferator-activated receptor α; lpl = lipoprotein lipase;* *i**l-10 =* *interleukin 10;* *il-**1β =* *interleukin 1β;* *nf-κb =* *nuclear factor kappa-B; tnf-α = tumor necrosis factor-α;*

**Table S2 The calculation formulas of the** **growth, feed utilization, and morphological indexes of fish fed experimental diets**

| **Indexes** | **The calculation formulas** |
| --- | --- |
| Weight gain rate (WGR) | (Final weight of fish - Initial weight of fish) / Initial weight of fish |
| Specific growth rate (SGR) | [ln (Final weight of fish) - ln (Initial weight of fish)] / Days of feeding ×100% |
| Feed conversion ratio (FCR) | Feed intake / (Final weight of fish - Initial weight of fish) |
| Survival rate (SR) | Number of final fish / Number of initial fish ×100% |
| Viscera index (VSI) | Viscera weight /Body weight × 100% |
| Hepatosomatic index (HSI) | Hepatopancreas weight / Body weight × 100% |
| Spleen index (SI) | Spleen weight / Body weight × 100% |
| Kidney index (KI) | Kidney weight / Body weight × 100% |
| Intra-peritoneal fat index (IFI) | Weight of intra-peritoneal fat / Body weight × 100% |
| Relative intestine length (RIL) | Intestine length / Body length × 100% |
| Condition factor (CF) | Body weight / Body length^3^ × 100% |
